# Study of the protective effects of cyanocobalamin on methotrexate induced nephrotoxicity in rats

**DOI:** 10.12688/f1000research.124081.1

**Published:** 2022-09-07

**Authors:** Rana Q. Abdulwahhab, Samara Muwafaq Ali Alabdali

**Affiliations:** 1 Pharmacology, College of Medicine, University of Baghdad, Baghdad, Adhamiya, 00964, Iraq

**Keywords:** Methotrexate, nephrotoxicity, cyanocobalamin, antioxidants, rat, histopathology

## Abstract

**Background: **Methotrexate (MTX) is a chemotherapeutic drug, used mainly in many cancerous stages, inflammatory and auto-immune diseases, but its use has been limited by its nephrotoxicity. Cyanocobalamin is a water-soluble vitamin possessing nephro-protective properties. The aim of this study was to investigate the effect of cyanocobalamin on the nephrotoxicity of methotrexate.

**Methods: **In the study 42 albino adult female rats were used, divided into six groups each containing seven rats (n=7). 1 ^st^ group: Control group (Negative control), 7 rats were injected intraperitoneally with 0.5ml/kg/day NS. Second group: 7 rats were injected intraperitoneally with a single dose of methotrexate (20 mg/kg) for 4 days. Third Group: 7 rats were given intraperitoneally cyanocobalamin at a dose (1.5 mg/kg/day) for two weeks, fourth, fifth, sixth group: 7 rats from each group were injected intraperitoneally with different concentrations of cyanocobalamin (0.5, 1, 1.5 mg/kg /day), respectively, for two weeks and MTX (20 mg/kg), which was injected only on day 11. On day 15, rats from all groups were euthanized, and blood samples were taken for biochemical tests, including evaluating serum urea and creatinine. The kidneys were extracted for histological investigation and evaluation of antioxidant (GSH) and oxidative stress (MDA) by using kidney tissue homogenates.

**Results:** This study revealed that kidney damage, produced by the MTX (group II), is manifested by significantly elevated (P<0.05) urea and creatinine. On the contrary, the cyanocobalamin groups (IV, V, VI) significantly (P<0.05) reduced urea and creatinine. Renal antioxidant defense systems, such as reduced glutathione depleted by MTX therapy, were restored to normal levels by cyanocobalamin. Furthermore, cyanocobalamin reduced oxidative stress (MDA) and histologically reduced renal tissue injury induced by MTX.

**Conclusions:** In conclusion, the study revealed that cyanocobalamin has a nephroprotective action upon MTX-induced renal damage in rats; cyanocobalamin may offer a protective effect, such as antioxidant action.

## Introduction

Methotrexate (MTX) is known as an antifolate drug and has been prescribed for over 60 years to treat different auto-immune disorders ^1^ and numerous types of cancers, used in combination with other anticancer drugs ^2^ and remains of great interest for researchers all over the world. ^2^

However, some adverse effects including nephrotoxicity triggered by MTX are severe, thus, assessment of effective drugs to overcome this problem are necessary. ^3^

MTX is one of the most common drugs induced nephrotoxicity, when used at high doses, having molecular features that favor crystal precipitation in the tubular lumens in a manner of slowing urine flow and decreasing urine pH. ^4^^,^^5^

Researchers reported that the mechanism of MTX inducing nephrotoxicity is as a result of oxidative damage by the formation of reactive oxygen species (ROS), then creating an imbalance between oxidants and antioxidants that are responsible for the adverse effect of MTX, such as nephrotoxicity. ^6^

Histologically in MTX–treated rats, kidneys showed: enlargement of Bowman capsule cavity, infiltration of lymphocytes, diminish of glomeruli size, an increase in blood cells count, and degeneration of tubules. ^7^^,^^8^

Like other drugs with nephrotoxic effects, MTX-induced renal function declination is clinically tested by observation of hematuria and deterioration in serum urea and creatinine level. Reproduction of such effects is done in research studies in animals after a single dose of MTX. ^9^

Many studies show that dietary antioxidants play a role in the protection of the kidney against MTX-induced nephrotoxicity. ^6^

Cyanocobalamin is considered a synthetic compound of vitamin B12. Vitamin B12 is known as a water-soluble vitamin which is synthesized by microorganisms in nature, and in the chemical aspect it is related to a class known as corrinoids. This name is derived from the cyanide group that is attached to molecule. ^10^

Vitamin B12 has several functions in methylation reactions in the body, acts as a cofactor in the form of methylcoblamin by the conversion of homocysteine to methionine and in the form of adenosylcobalamin in the transformation of methylmalonyl-CoA to succinyl-CoA. Both of these chemical reactions are important for cell division and growth, ^11^ Vitamin B12 plays a role in erythropoiesis and healthy neurological functions, ^12^ and it proved useful in the treatment of many diseases associated with inflammatory and oxidative stress. ^13^

The FDA have stated that cyanocobalamin is safe, there is no toxicity reported when cyanocobalamin given to animals, even at several thousand times their nutritional requirements. ^14^

The objective of this study is to determine the protective effects of cyanocobalamin administration on methotrexate-induced nephrotoxicity in rats using biochemical and histopathological studies in renal tissue of rats.

## Methods

### Ethical approval

We have received ethical approval for work on experimental animals from the **Researcher Ethics Committees** at Department of Pharmacology and College of Medicine/University of Baghdad. On 7 November 2021 *via* ethical letter (approval number 1455). This study is reported in line with the ARRIVE guidelines. ^40^

### Chemicals

All chemicals used in this experimental study were of the highest available purity, and no purification was necessary. Vitamin B12 GERDA (1000 mcg/4 ml) (GERDA ^®^1000, batch: H060) was purchased from GERDA, France. Methotrexate ampule (50 mg/2 ml) Methotrexate Mylan 50 mg/2 ml (batch/LOT 5103) was purchased from Mylan – Merck generiques, Italy, pellet food patch number 3218 from **Mazuri** ^®^ **food** pellets (Mazuri Primate Diet, Special Diet Foods Ltd., UK).

### Experimental animals

The study was carried out on 42 adult female albino rats. The sample size of n=7 per group was calculated based on previous research, inclusion criteria include (ages ranged from 2-3 months, sex: female and body weight ranged from 150-250 grams. In this study there were no exclusion criteria, the animals used were were obtained from the same source, at the same time, there is no previous procedure performed on these rats before the experimental study.

The rats were obtained from the Animal House of the Iraqi center of cancer and genetic research/Almustansria University. They remained for one week, to be adapted without intervention in a climate-controlled environment with appropriate temperature (22-25 c) and synthetic 12:12 hours in the light-dark cycle, rats received pellet food and tap water/ad libitum.

The human care of the animals was according to international guidelines for the care and use of laboratory animals, All of these efforts were made to reduce the number of rats and their suffering, These efforts included: 1. constant checking of animal house air conditioning with a standard free access to tap water/ad libitum; 2. cleaning the animal house and cages; 3. During the experimental careful handling and when given the dose of drug injection as quickly as possible and given the dose to each rat separated from other; 4. used the effective anesthetic dose.

When the period of adaptation was over, the weight of the rats was recorded and they were randomly divided and placed in one cage per group, the name of the group was then recorded in each cage.

In total, 42 rats were randomly (simple randomization) divided into six group (n=7) All groups of animal, treated in the same house under same environment. The experimental procedure was done blindly, daily between 8 am -12 pm for 15 days.

Six animal groups were made in this study (7 animals/each group) as follows:

**Group I** (control): 7 rats were treated with 0.5 ml normal saline once daily by intraperitoneal (I.P.) injection for 14 days and it was served as negative control.

**Group II (MTX**) **:** 7 rats were treated with a single I.P. dose of methotrexate (MTX) (20 mg/kg) afterward, a single I.P. injection of 0.5 ml was administered. normal saline for 4 days. This group served as positive control. ^15^

**Group III (1.5 mg of vit b12):** 7 rats were treated with vitamin B12 (1.5 mg/kg/rat/day) once daily by I.P. for two weeks.

**Group IV (0.5 mg vit b12 +MTX):** 7 rats were treated with vitamin B12 (0.5 mg/kg/rat/day)) once daily by I.P. for two weeks + 20 mg/kg I.P. of MTX injected only at day 11. ^16^

**Group V (1 mg vit b12 +MTX):** 7 rats were treated with vitamin B12 (1 mg/kg/rat/day) once daily by I.P. for for two week + 20 mg/kg I.P. of MTX injected only at day 11.

**Group VI (1.5 mg vit b12 +MTX):** 7 rats were treated with vitamin B12 (1.5 mg/kg/rat/day) once daily by I.P.for two week + 20 mg/kg by I.P. of MTX injected only at day 11. ^16^

At the end of this experiment on day 15 the rats were euthanized by the use of xylazine (10 mg/kg) and ketamine (75 mg/kg) ^17^ both are commonly used for this purpose (this is following the ethical standards and ARRIVE guidelines and this type of euthanization is normally performed in most acute toxicity tests). Kidney tissue samples were taken for biochemical ^37^ and histopathological studies. ^38^

### Sample collection and tissue preparation

After euthanasia of the animals, blood was collected by intracardiac puncture. The clot was isolated with a glass rod and then centrifuged (CGOLDENWALL 80-2 Electric Lab Centrifuge) at 3000 rpm for 15 minutes, and the supernatant was used to evaluate urea and creatinine as parameters of kidney function. ^37^ The abdomen was cut with a scalpel blade, then the kidney was extracted and then stored in 10% formalin (Pan Reac APPliChem, Cat NO:A0877.100).

A small portion of the kidney was cut and weighed on an electrical weighing scale (Redwag, model: AS220.R1). A sample of almost 0.4 gram weight was converted to homogenizer (Omni TH, Cat NO, 51-001) and mixed with 1 ml of ice cold phosphate buffered saline and centrifuged for 15 minutes at 5000 rpm. Subsequently, the resulting supernatant was isolated and stored at -20° C until used for the determination of the MDA and GSH biomarkers. ^37^

### Investigated parameters

**Histological examination of the kidneys**

**Figure 1.**
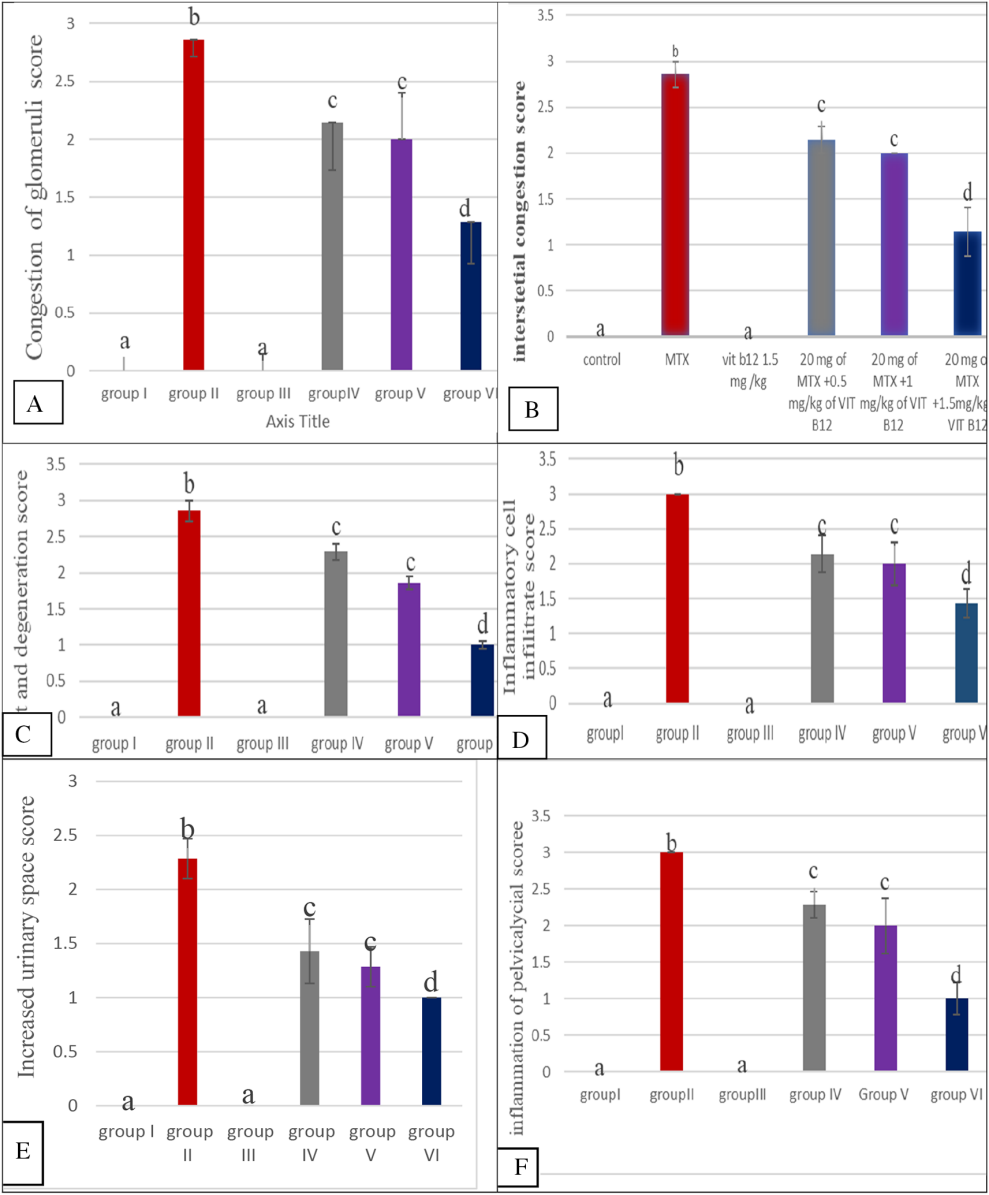
The histological scores of all groups. Values are mean±SEM for seven rats in each group, values expressed in nonidentical letters (a, b c, d) are significantly different at the P value (P<0.05) (N=7).

Histopathological studies were performed, kidney tissue samples were fixed in 10% formalin, then graded in ethanol, dehydrated, and embedded in paraffin. Kidney sections were cut with a microtome (HHQ-1508R Rotary **Microtome**, **China**) set at a thickness of 3-4 μm; mounted on clear glass, then the kidney tissue was stained with Hematoxylin and Eosin (H&E). Then the kidney tissue sections were examined and evaluated by the histopathologist using light microscopy at 10× and 40× (Viola Mc 20i, Micros ^®^ Austria). ^18^ An overall score of the severity of damage to kidney tissue was assessed in stained tissue sections by scoring each of the following histopathological observations as shown in Figure 1A. Congestion of glomeruli B. Congestion of interstitial C. Cast and degeneration D. Inflammatory cell infiltrate E. Increased urinary space F. Inflammation of the Pelvicalyceal area.

Where, 0=none (no damage); 1=mild; 2=moderate; 3=severe. A total of field of section was examined for all animals in each group ^19^^,^^38^

### Serum analysis

**Determination of serum creatinine and urea level**

The quantitative assay of creatinine using the jaffe method was used. The quantitative assay of creatinine by the jaffe method is based on the reaction of creatinine with sodium picrate, and creatinine reacts with alkaline picrate forming a red-color complex. The intensity of the formed color is proportional to the creatinine concentration within the sample and the creatinine level can be calculated by measuring the absorbance at 490 nm. ^20^ This method was performed using a creatinine kit (Cat. NO D500, Spinreact, Espana), while urea was measured quantitatively using the enzyme method described below, ^21^ using a urea kit (Cat.NO p406, Spinreact, Espana).

**Enzymatic method**

Quantitative determination of serum urea by depend on enzymatic reaction in which the urea within the sample is hydrolyzed enzymatically into ammonia (NH4 +) and carbon dioxide (CO2). Ammonia ions formed reacts with α-ketoglutarate in a reaction catalyzed by glutamate dehydrogenase (GLDH) with simultaneous oxidation of NADH to NAD+:

The decrease in NADH concentration is proportional to the concentration of urea within the samples, and the level of urea can be calculated by measuring the absorbance at 340 nm. Calculation of urea (mg/dl) within serum samples = (∆A) Sample × 50 (Standard conc.) = mg/dL urea in the sample.

## Determination of malondialdehyde (MDA) and reduced glutathione (GSH) within the kidney homogenate of the rat

Malondialdehyde and reduced glutathione levels were measured by the enzyme-linked immunosorbent assay (ELISA) sandwich technique. ^22^

The MDA content in the kidney tissue homogenate was quantitatively estimated by the MDA kit based on the ELISA method according to the manufacturer's instructions (Cat. No. E0156Ra, Bioassay Technology Laboratory, China) and Rat reduced glutathione ELISA kit (Cat. No E1443Ra, Bioassay technology laboratory, China), as written on the manufacturing protocol.

The content of MDA and GSH in kidney tissue homogenate samples can be measured by comparing the optical density of the samples, with the standard curve. Level of MDA is expressed as nmol/mL, and level of GSH is expressed in Ng/ml.

### Elisa MDA kit procedure

We prepared all reagents and chemicals tokit in the kit box (and stored at room temperature, then we were Add 40 μl sample to sample wells and then add 10 μl anti-MDA antibody to sample wells, then 50 μl streptavidin-HRP to sample wells. Then we mixed them well and Incubated 60 minutes at 37°C. We removed the sealer and cleaned the plate 5 times with wash buffer. Then the product was placed in automated washing (biotek ^®^ ELX 600-biotek Co. – USA), then we determined the absorption of the plate using Elisa reader equipment (biotek ^®^ ELX 600-biotek Co. – USA) the absorption is directly proportional to the concentration, which is then estimated by comparing the reading with the control group.

### GSH procedure

This was the same as the MDA procedure.

### Statistical analysis

Data was expressed statistically as the mean±SEM, (standard error of mean) and the significance of the differences among various groups was decided by one-way analysis of variance (ANОVA), then by least significant difference (LSD) test. The statistical package for the social sciences (SPSS) version 26 was used. Differences were deemed statistically significant at the P-value less than 0.05 level. ^23^

## Results

### Effect of cyanocobalamin on GSH contents within kidney tissue homogenate of rats

A nonsignificant difference was observed between the cyanocobalamin (group III) and control groups (group I). ^37^

**Figure 2.**
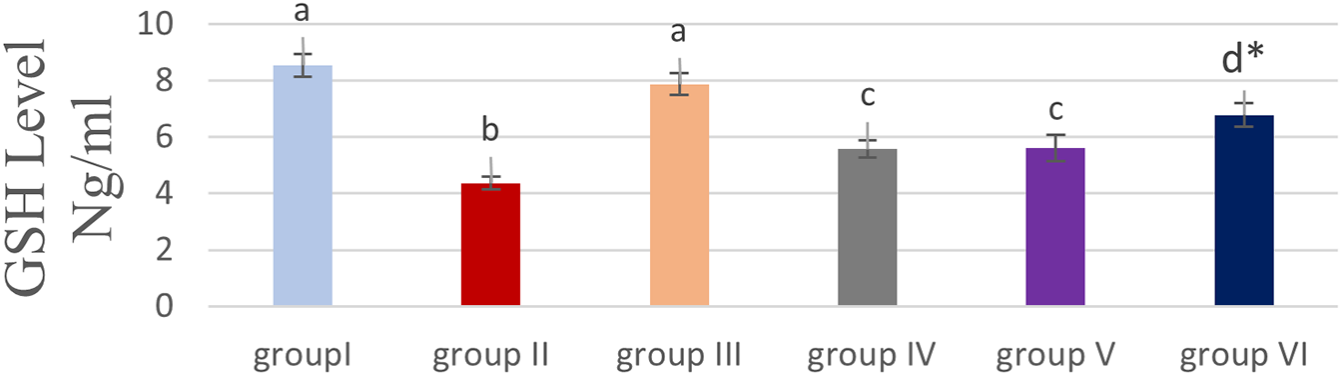
Bar chart showing the effect of cyanocobalamin on glutathione (GSH) contents in the kidney tissue homogenate of rats.

While the level of GSH in the renal tissue homogenate decreased significantly decreased (P<0.05) in both groups IV and V, it was highly significantly decreased in group VI compared to the group treated with MTX treated group (P<0.01) as shown in Figure 2.

### Effect of cyanocobalamin on Malondialdehyde (MDA) Levels within the kidney tissue Homogenate of Rat

**Figure 3.**
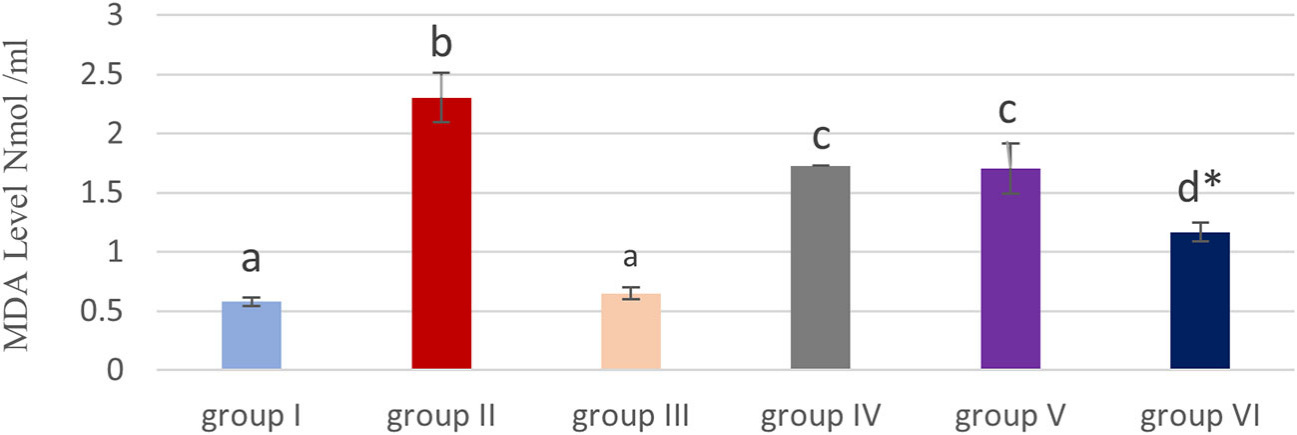
Bar chart showing effect of cyanocobalamin on malondialdehyde (MDA) contents in the kidney tissue homogenate of rats.

Table 1 and Figure 3 show that, compared to the control and cobalamin groups (I, III), the MDA level was significantly (P<0.05) elevated in the methotrexate group (II).

**Table 1.** Effect of cyanocobalamin on levels of malondialdehyde (MDA) and glutathione (GSH) within the kidney tissue homogenate of rats.

Group N=7/group	Malondialdehyde (MDA) nmol/ml	Glutathione Ng/ml
Group I (control) /I.P. injected with 0.5 ml/kg/day normal saline	0.57829±0.038840 ^a^	8.53457±0.411815 ^a^
Group II/MTX I.P. 20 mg/kg	2.30229±0.208563 ^b^	4.35600±0.224845 ^b^
Group III/B12 1.5 mg/kg for 14 days	0.64900±0.049109 ^a^	7.87243+0.396563 ^a^
Group IV (20 mg of MTX +0.5 mg/kg of VIT B12 for 14 days)	1.72586±O.285667 ^c^	5.55929±0.308215 ^c^
Group V(20 mg of MTX +1 mg/kg of VIT B12 for 14 days)	1.70429±0.211100 ^c^	5.59300±0.463932 ^c^
Group VI (20 mg/kg of MTX +1.5 mg/kg of vit b12 for 14 days)	1.16757±0.077618 ^d^ ^*^	6.77829±0.414523 ^d^ ^*^

Each value represents the mean±standard error of the means (SEM) (N=7/Group). Values expressed in non-identical letters (a, b,c,d) are significantly difference (P<0.05).

* Highly significant difference from MTX P<0.01.

Non-significant differences were detected among the cyanocobalamin (group III) and control groups (group I).

Although MDA levels in renal tissue homogenate (P<0.05) significantly in the IV & V groups, and decreased significantly in group VI compared to the the MTX treated group (P<0.01).

### Effect of cyanocobalamin on plasma urea (U) and creatinine (CR) level

**Figure 4.**
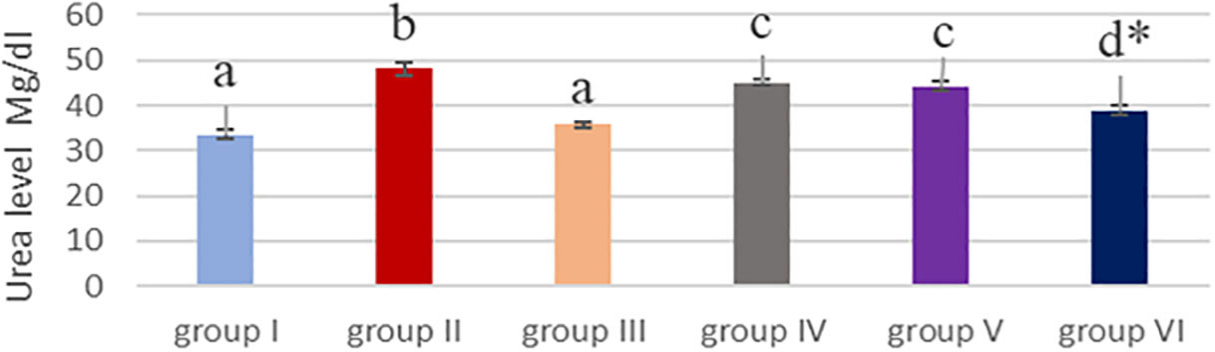
Bar chart showing effect of cyanocobalamin on plasma urea level.

**Figure 5.**
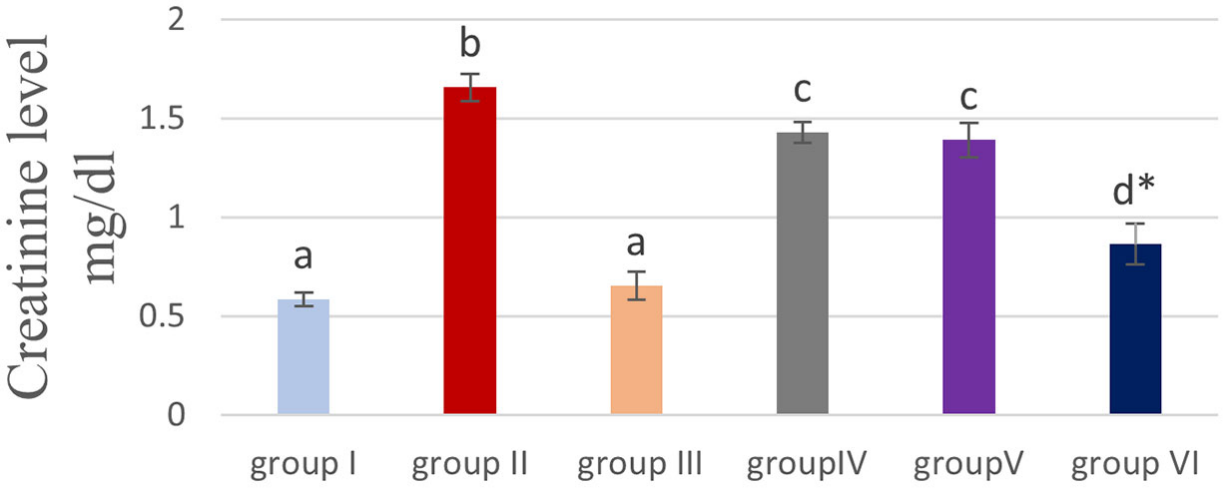
Bar chart showing the effect of cyanocobalamin on plasma creatinine level.

Table 2 and Figures 4 & 5 showed that the methotrexate-treated rat group (II) produced a significant elevation (p <0.05) of the plasma level of urea and creatinine, compared to the corresponding levels of the control group (I) and the cobalamin group (II).

**Table 2.** Effect of cyanocobalamin on plasma urea and creatinine level.

Group N=7/group	urea mg/dl	Creatinine mg/dl
Group I (control) /I.P. injected with o.5 ml/kg/day normal saline	33.557±0.9198 ^a^	0.586±0.0340 ^a^
Group II/MTX I.P. 20 mg/kg	48.071±1.4276 ^b^	1.657±0.0685 ^b^
Group III/B12 1.5 mg/kg for 14 days	35.757±0.5871 ^a^	0.653±0.0706 ^a^
Group IV (20 mg of MTX +0.5 mg/kg of VIT B12 for 14 days)	45.143±0.5084 ^c^	1.429±0.0522 ^c^
Group V (20 mg of MTX +1 mg/kg of VIT B12 for 14 days)	44.286±1.0169 ^c^	1.390±0.0879 ^c^
Group VI (20 mg/kg of MTX +1.5 mg/kg of vit b12 for 14 days)	38.886±1.1365 ^d^ ^*^	0.864±0.1036 ^d^ ^*^

Each value represents mean ± standard error of means (SEM) (N=7/Group), Values expressed in non-identical letters (a, b, c, d) are significantly difference (p<0.05).

* Highly significant difference from MTX P<0.01.

Non-significant difference (P>0.05) of the serum of urea and creatinine was found between the control group (I) and cobalamin group (III).

Rats treated with different concentrations of cyanocobalamins in group IV and group V produced significantly decreased serum urea and creatinine levels levels (P<0.05), while group VI produces highly significantly decreased serum urea levels compared to MTX-treated group II (P<0.01).

### Histological findings

Histopathological changes were examined to assist the finding of biochemical markers. ^38^^,^^39^

**Figure 6.**
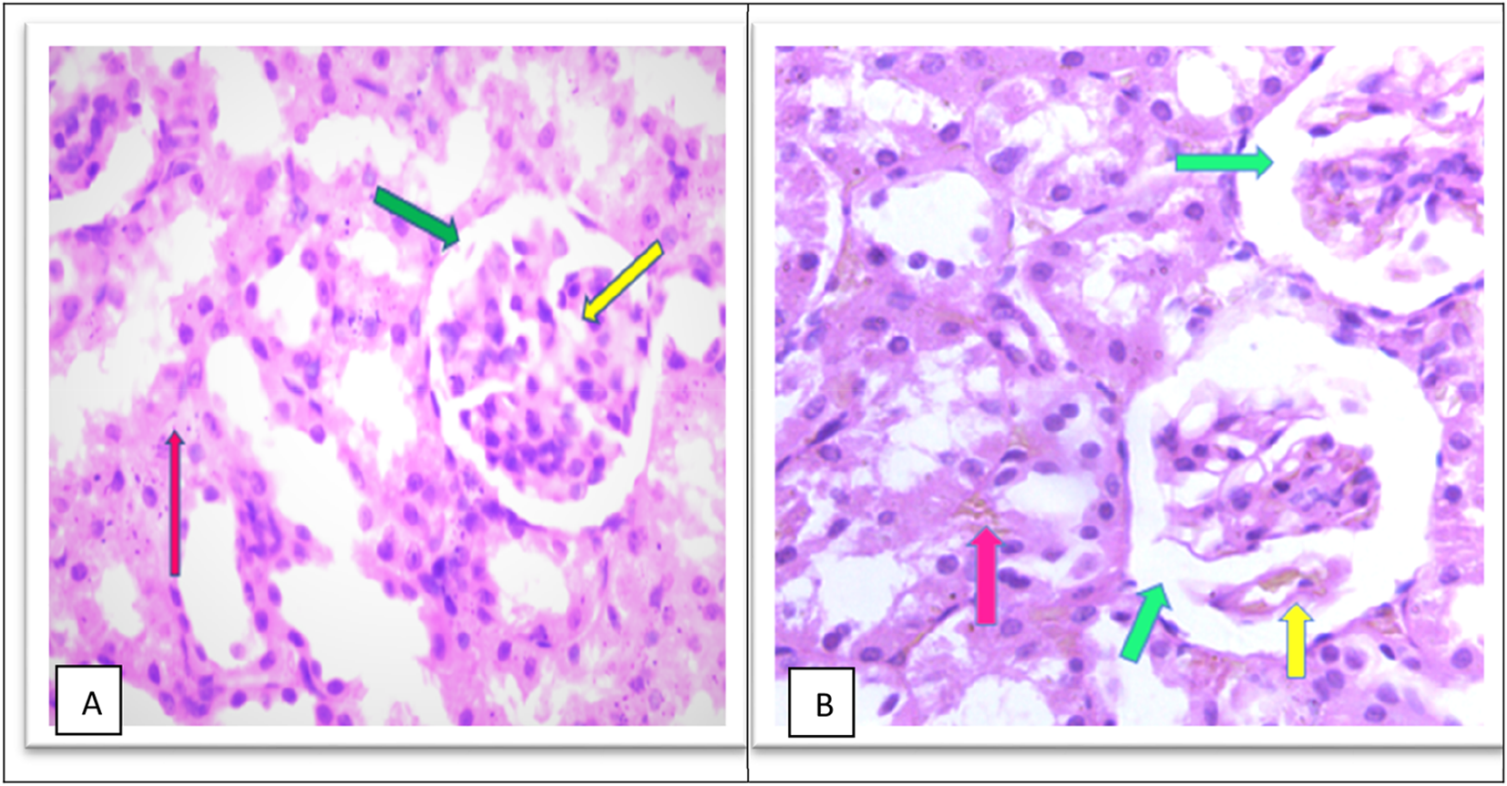
(A) section of kidney control group (Group I) of rat shows a Normal looking cortex, the glomeruli are with in normal (yellow arrow) no congestion of their capillaries, the urinary space is not dilated (green arrow), and the interstitial shows no congestion (pink arrow) (H&E ×40). (B) The kidney tissue section of rat treated with methotrexate for 4 days (group II) shows severe glomeruli congestion (Yellow arrow) and atrophy with widening of the urinary space (green arrow), (green arrow), severe congestion is also observed in the interstitial (pink arrow) (H&E ×40).

The MTX –treated group (II) kidneys revealed obvious variations and injuries, characterized by severe congestion of both interstitial and glomeruli, moderate increased urinary space causing shrinkage in the glomerular size as shown in Figure 6B and a high number of hyaline casts in the medullary region with degeneration of tubules as shown in Figure 6C, severe inflammation of inflammatory cell infiltrate as shown in Figure 6D and pelvicalycial system as shown in the as shown in Figure 6E in the *Extended data.* ^39^

**Figure 7.**
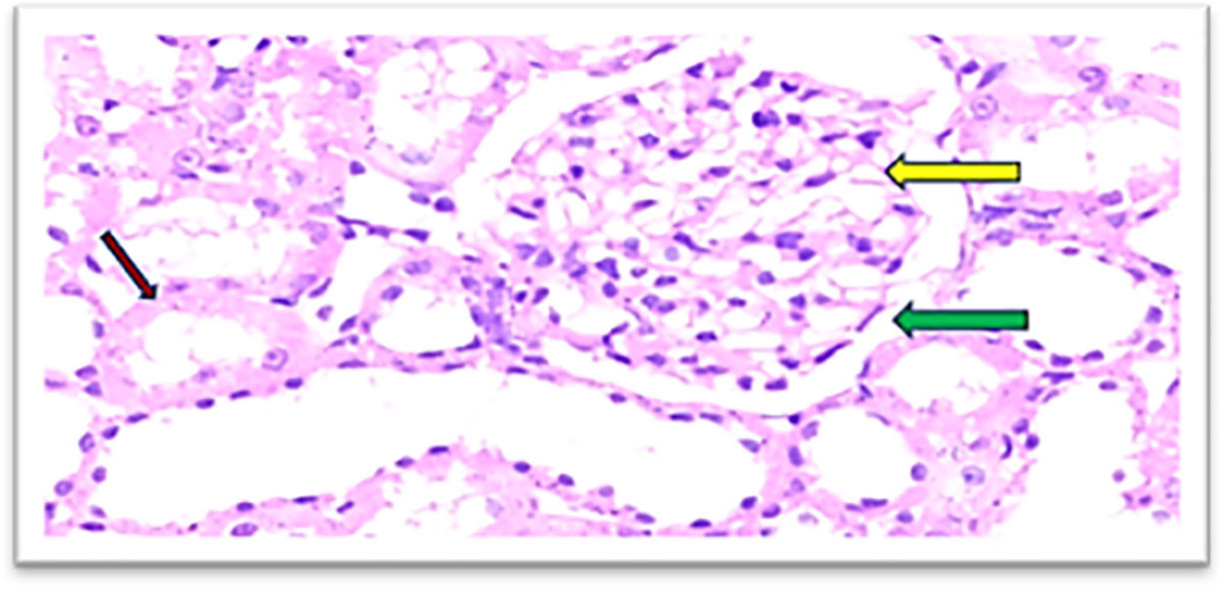
Section of the kidney tissue of rat treated Cyanocobalamin for 14 days (Group III) show A normal glomerulus (yellow arrow) and the urinary space is within normal limits (Green arrow), the interstitial shows no congestion (Pink arrow) (H&E, ×40).

Furthermore, the cobalamins group (group III) show a normal glomerular cellularity (no congestion) and the urinary space is within normal limits. The interstitial does not show congestion; the tubule does not show degeneration and no inflammatory infiltrate as shown in Figure 7, similar to the control group (group I), which shows a normal looking cortex; the glomeruli have normal cellularity, no capillary congestion, the urinary space is not dilated; the interstitial does not show congestion, no inflammatory infiltrate, there is no tubular cell degeneration, and no casts. The pelvicalyceal system is not shown here, as shown in Figure 6A.

**Figure 8.**
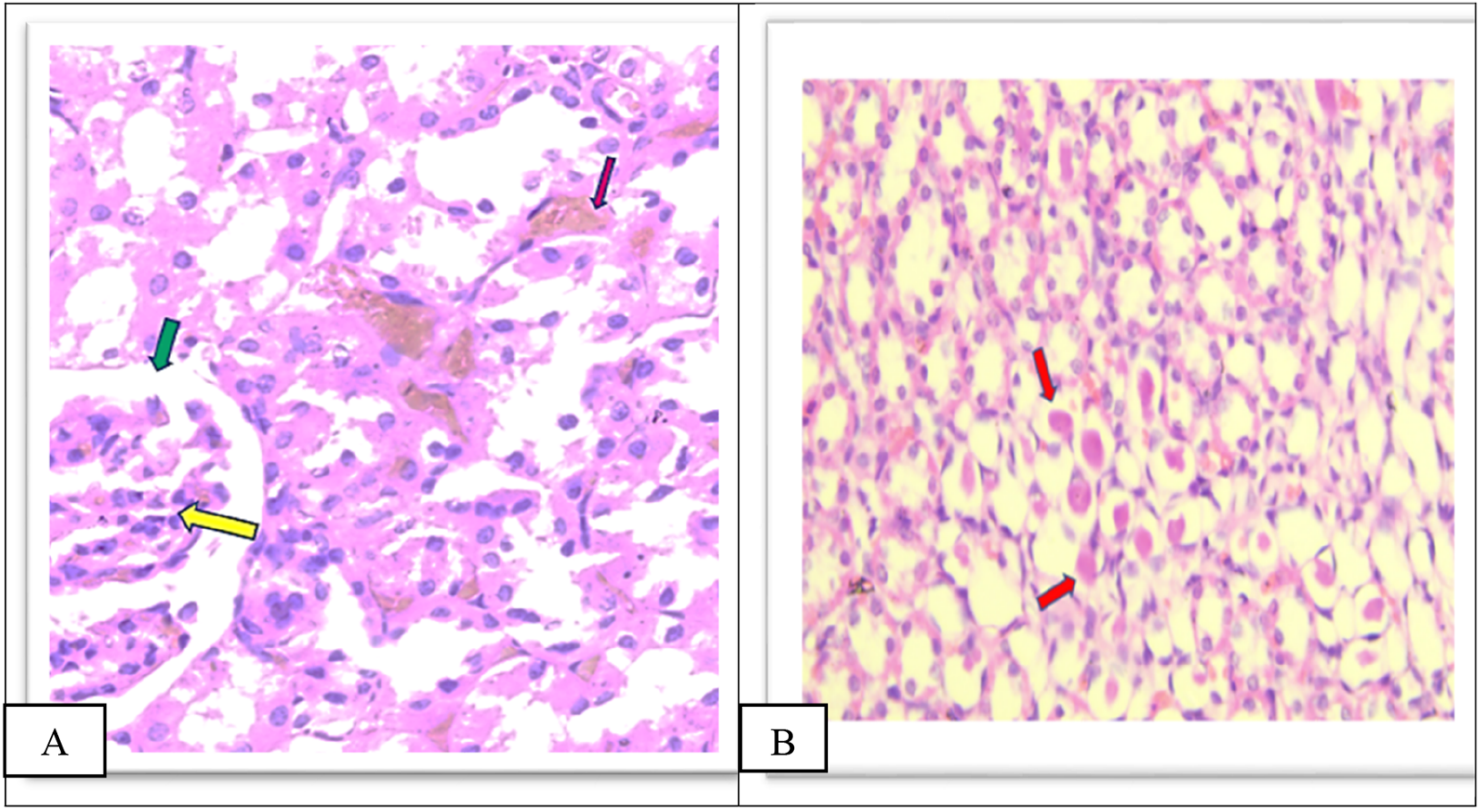
A. Section of the kidney tissue of rat (Group IV) show moderate congestion of interstitial (Pink arrow), moderate congestion of glomeruli (yellow arrow) and moderate increased of urinary space (Green arrow) (H&E, ×40). B. The section of rat kidney tissue (Group IV) shows degeneration and moderate amount of hyaline cast formation (Red arrow) (H&E, ×40).

Group IV showed a moderate increase in urinary space, moderate congestion of the glomeruli and interstitial as shown in Figure 8A, a moderate amount of hyaline cast formation and degeneration as shown in Figure 8B, moderate inflammation of the pelvicalycial system Figure 8c, moderate infiltrate of inflammatory cells of the interstitial Figure 8d as shown in the *Extended data.* ^39^

**Figure 9.**
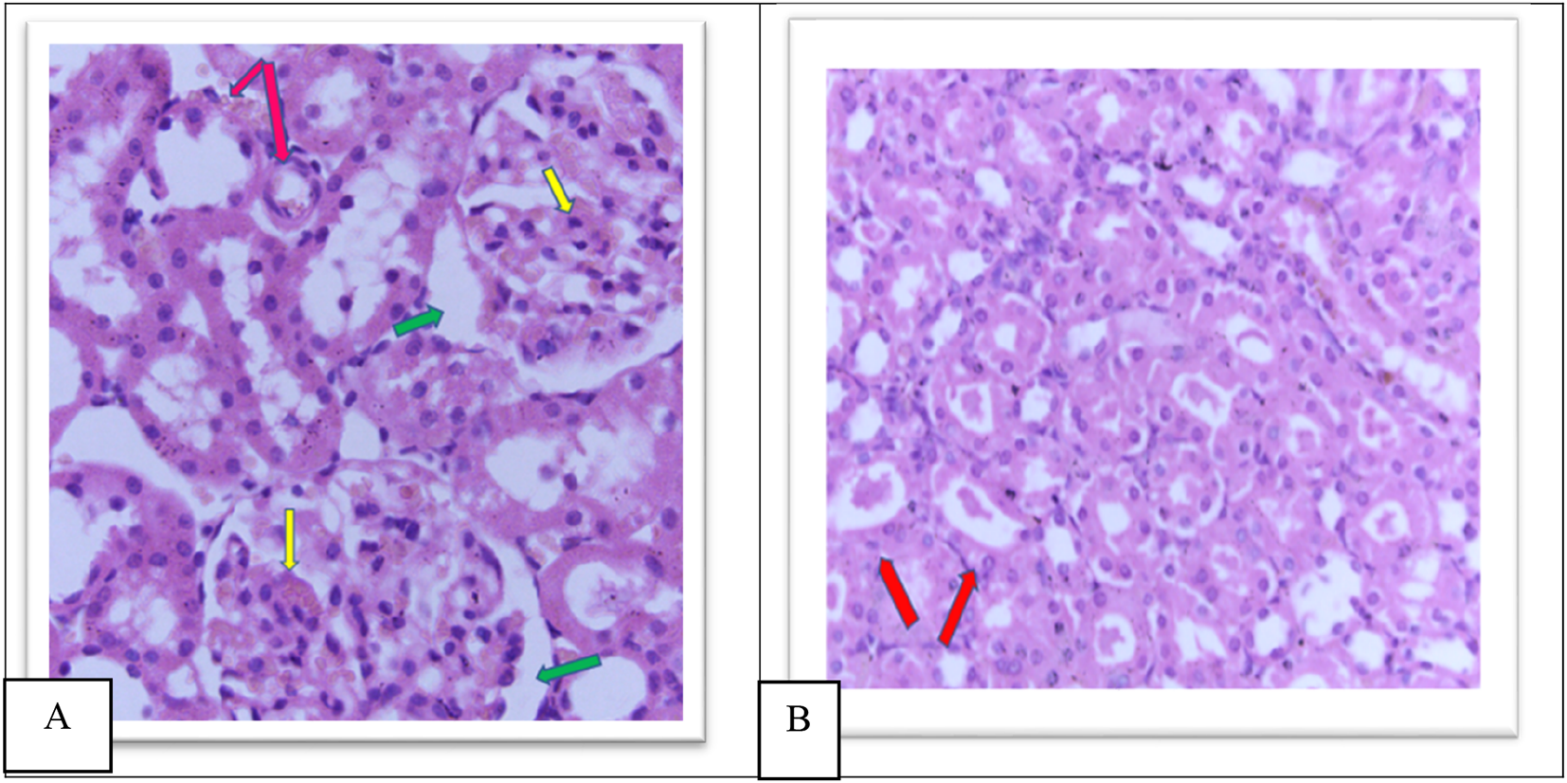
A. Section of the kidney tissue of rat (Group V) Show moderate congestion of interstitial (Pink arrow), moderate congestion of glomeruli (yellow arrow) and moderate increased of urinary space (Green arrow) (H&E, ×40). B. The section of rat kidney tissue (Group V) shows a moderate tubular degeneration and a moderate formation of hyaline casts with in tubular lumens (red arrow) (H&E, ×40).

Group V showed a moderate increase in urinary space, moderate congestion of glomeruli and interstitial as shown in Figure 9A, moderate amount of hyaline cast in the tubular lumen and degeneration as shown in Figure 9B, moderate inflammation of the pelvicalycial system 9C, moderate inflammatory cell infiltrate of interstitial as shown in Figure 9D as shown in the *Extended data.* ^39^

**Figure 10.**
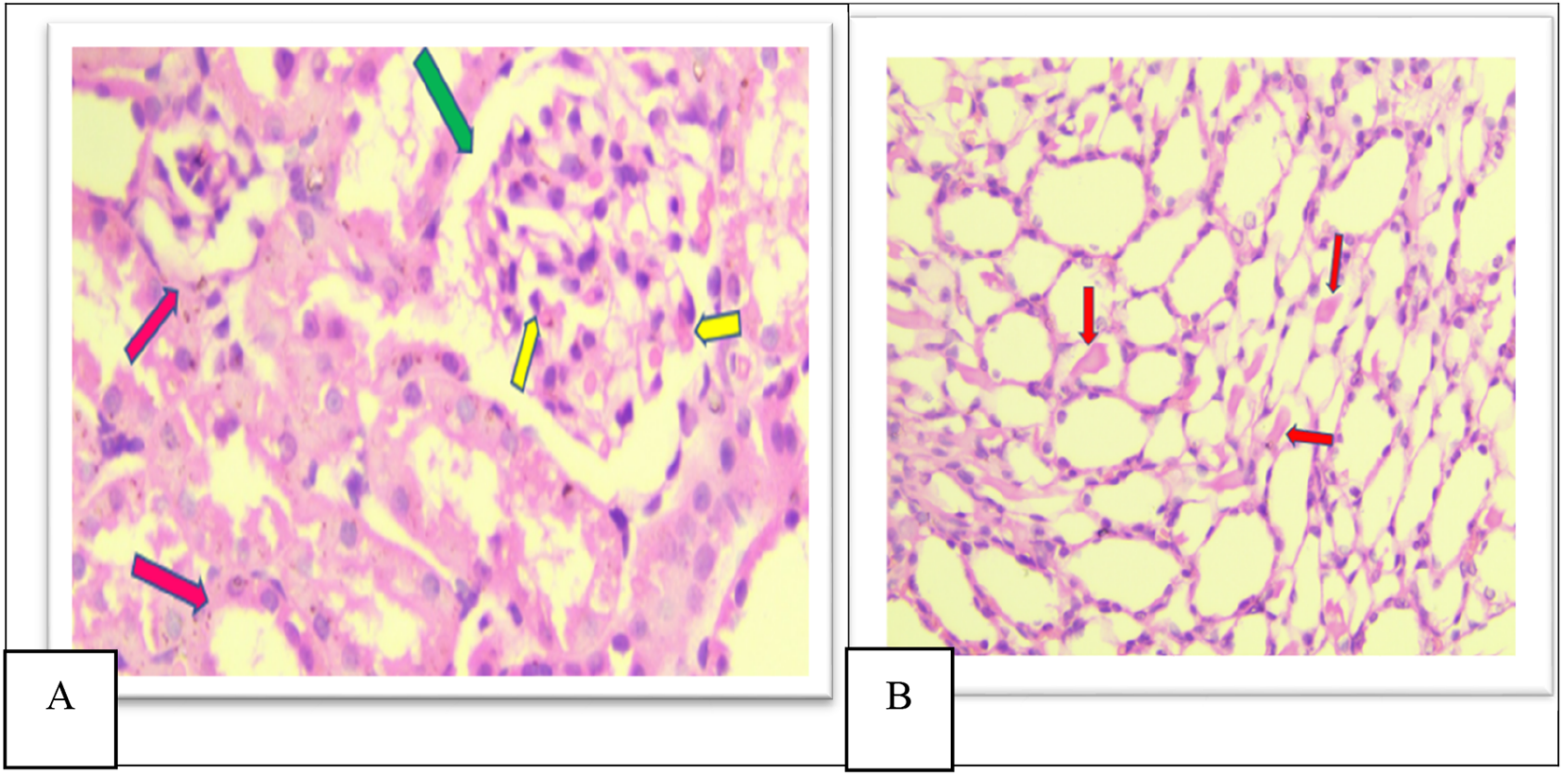
A. Section of the kidney tissue of rat (Group VI) Shows mild congestion of glomeruli (Yellow arrow) the interstitial space shows mild congestion and degeneration (Pink arrow) and mild increased urinary space (Green arrow) (H&E, ×40). B. Section of the kidney tissue of the rat (Group VI) Shows that a few casts (Red arrow) are seen within tubular lumens (H&E, ×40).

Furthermore, in group VI there was a significant decrease in damage in all histopathological parameters compared to other groups (II, IV, and V), There is mild glomerular congestion and increased cellularity, the interstitial space also shows mild congestion and degeneration, mild increased urinary space as shown in Figure 10A, Few casts are seen within tubular lumens as shown in Figure 10B, mild inflammatory infiltrate in the pelvicalycial system as shown in Figure 10c, mild inflammatory cell infiltrate of the interstitial as shown in Figure 10d in the *Extended data.* ^39^

## Discussion

The renal system plays a vital role in maintaining extracellular environment homeostasis, mainly detoxification and elimination of toxic drugs and their metabolites. Nephrotoxicity related to toxic medications, especially chemotherapeutic drugs, is common and represents approximately 20% of all renal failure cases. ^24^

This study focused on the protective effect of cyanocobalamin on MTX-induced renal damage in rats by reducing Cr and urea levels and repairing histopathological damage to the kidney.

The present data established that the activities of vit B12 against MTX-induced renal damage in rats could be successful by suppressing oxidative stress by reducing MDA levels and increasing GSH levels.

There were significant elevations in creatinine and urea levels in the MTX treated group (group II), compared to the levels in the the control rats (group I) and the the cyanocobalamin treated group (group III) (P<0.05).

Researchers indicated the main pathway for MTX elimination by kidney. Furthermore, the administration of high doses of MTX causes higher drug concentrations in plasma and urine, at an acute and extremely toxic dose the elimination of MTX is delayed execution, then increases the serum creatinine level, and thus is one of the biomarkers of renal failure, this is consistent with the present study. ^25^^,^^26^

Group IV, V, and VI resulted in a significant ( *P*<0.05) reduction (P <0.05) in plasma urea and creatinine levels, compared to those levels in group II. That is consistent with the finding of Alshanwani *et al.,* ^24^ which showed that vitamin B12 improved creatinine clearance levels and renal blood flow, thus, this can reduce creatinine and blood urea nitrogen. ^24^

In the current study, a non-significant difference was found (p >0.05) within the content of MDA of kidney tissue homogenate among the control (group I) and cyanocobalamin (group III), which is in line with a previous animal study. ^13^

MTX treated rats (group II) demonstrated significant increases in MDA levels in group II, compared to control (group I) and cyanocobalamin (group III) which is consistent with previous studies. ^27^^,^^28^

Increased levels of MDA in the methotrexate group might be related to oxidative stress, which likely stores the leukocyte in tissues by increased the level of ROS. ^29^ Active leukocytes release many chemical enzymes, such as protease, elastase, and myeloperoxidase, resulting in more free radicals.

In addition, reactive oxygen species produce changes in permeability of both endothelial and epithelial cells. It can produce tissue injury, caused by MTX-prompted OS, by interfering with unsaturated form fatty acids of cell membranes, nucleic acid and sulfhydryl bonds. MTX therapy can indirectly secrete mitochondrial enzymes that lead to damaged mitochondria, and a decrease in antioxidant action. ^8^

In this study, groups IV, V and VI produced a significant ( *P*<0.05) reduction (P <0.05) in the level of MDA, compared to the group II treated with MTX.

These results are due to the antioxidant function of Cyanocobalamin by inducing the activity of methionine synthase, interacting with ROS, and avoiding glutathione. ^13^^,^^30^

Glutathione (GSH) is a non-enzymatic antioxidant; preserving reduced glutathione in the cell is essential to improve the immune system and protect the body from diseases. ^31^^,^^32^

GSH act as reducing agents that are important for conserving enzymes and antioxidants in the form of an active state. This mechanism is important to provide protection from cytotoxic agents, free radicals, and oxidative damage. ^33^

In this study, the MTX group (group II) produced a significant reduction in the level of GSH, compared to the control group (group I) which is in line with a previous study. ^3^

This result may be related to the mechanism of OS generation by MTX by inhibiting cellular NADPH (nicotinamide adenine dinucleotide phosphate). Through the pentose phosphate cycle, the glutathione reductase enzyme NADPH is used as a reducing agent for cellular GSH (primary antioxidant). The reduction of cellular GSH by MTX decreased the systemic antioxidant action. ^34^

Group IV, V, and VI produced a significant (P<0.05) raise in GSH levels compared to those levels in groups II, these effects could be explained by the fact that cyanocobalamin has antioxidant properties consistent with the findings of Padmanabhan *et al.,* which showed that supplementation with cyanocobalamin was confirmed by increased GSH, this institutes the main intracellular antioxidant and reduces oxidative stress. ^33^

However, histological analysis revealed that, unlike the control group (group I), the methotrexate group (group II) had destructive effects, where kidney sections of such rats showed moderate increased urinary space causing shrinkage in the glomerular size, forming a high number of hyaline casts in the medullary region, with degeneration of tubules, congestion of both interstitial and glomeruli. The glomerular show reduced cellularity, severe inflammation of the inflammatory cell infiltrate, and pelvicalyceal system. This is similar to the findings of other previous studies. ^7^^,^^8^

The exact mechanism of MTX nephrotoxicity was unclear. However, some studies found that the main factor in MTX-related tissue injury was oxidative damage, with successive generation of more free radicals, the role of oxidative stress has been reported in MTX-induced nephrotoxicity. ^35^

Moreover, in groups IV, V and VI, there was a significant decrease in damage in all histological parameters (Expansion of bowman space, Congestion of glomeruli, Congestion of interstitial, Cast and degeneration, Inflammatory cell infiltrate, Inflammation of pelvicalycial system) (P<0.05), as compared to group II. These findings are similar to those of Saeed *et al* ^36^; where the protective effect of vitamin B12 against nephrotoxicity was observed by histopathological examination. ^36^

These results were considered an index of the defensive effect of cyanocobalamin on the kidney damaged by the methotrexate drug, the protective action of vitamin b12 has been previously reported in experimental studies. ^24^

## Conclusion

This study demonstrates the beneficial effects of cyanocobalamin on MTX-induced nephrotoxicity. Data suggest that the protective effect of cyanocobalamin is through the amelioration of oxidative markers (MDA) in kidney homogenate; the improvement of antioxidants (GSH), compensating for the increase in urea, creatinine, and the improvement of histopathological lesions of the kidney of rats. These results will make a new technique of protection from nephrotoxicity by MTX therapy, by administration of cyanocobalamin.

## Data availability

### Underlying data

Zenodo: Study in the protective effects of cyanocobalamin on Methotrexate induced Nephrotoxicity in rats. https://doi.org/10.5281/zenodo.6842130. ^37^

This project contains the following underlying data:

- Z.xlsx (biochemical test plasma of urea and creatinine, antioxidant enzyme (GHS) and oxidative stress marker (MDA))

Zenodo: Study in the protective effects of cyanocobalamin on Methotrexate induced Nephrotoxicity in rats. https://doi.org/10.5281/zenodo.6970347. ^38^

This project contains the following underlying data:

- Histopathological score.xlsx

### Extended data

**Zenodo**: Study in the protective effects of Cyanocobalamin on Methotrexate induced Nephrotoxcity in rats. https://doi.org/10.5281/zenodo.7008082. ^39^

This project contains the following extended data:

‐ Supplementary data.docx (histopathological images of kidney section of all six group (n=7))

## Reporting guidelines

Zenodo: ARRIVE checklist for ‘Study in the protective effects of cyanocobalamin on Methotrexate induced nephrotoxicity in rats’. https://doi.org/10.5281/zenodo.6982116. ^40^

Data are available under the terms of the Creative Commons Attribution 4.0 International license (CC-BY 4.0).
