# Supplementary material for: Study of the protective effects of cyanocobalamin on methotrexate induced nephrotoxicity in rats
Source: F1000Res. 2022 Oct 31;11:1012. Originally published 2022 Sep 7. [Version 2] doi: 10.12688/f1000research.124081.2 (PMC9641110; doi:10.12688/f1000research.124081.2)
Supplement: Peer review history

# Peer review: Reviewer response for version 2

**DOI:** 10.5256/f1000research.139755.r154541

**Published:** 2022-11-07

**Authors:** Israa Albanaa

**Affiliations:** 1 College of Pharmacy, Department of Pharmacology and Toxicology, University of Mosul, Mosul, Iraq

I have no further comments to make. You can accept the article for publication.

Is the work clearly and accurately presented and does it cite the current literature?

Partly

If applicable, is the statistical analysis and its interpretation appropriate?

Partly

Are all the source data underlying the results available to ensure full reproducibility?

Partly

Is the study design appropriate and is the work technically sound?

Yes

Are the conclusions drawn adequately supported by the results?

Yes

Are sufficient details of methods and analysis provided to allow replication by others?

Yes

Reviewer Expertise:

Pharmacology and toxicology

I confirm that I have read this submission and believe that I have an appropriate level of expertise to confirm that it is of an acceptable scientific standard.

# Peer review: Reviewer response for version 2

**DOI:** 10.5256/f1000research.139755.r154540

**Published:** 2022-11-01

**Authors:** Hasan Alhaddad

**Affiliations:** 1 Moffitt Cancer Center, Toledo, OH, USA

The authors addressed my comments. I recommend this article for the publication.

Is the work clearly and accurately presented and does it cite the current literature?

Yes

If applicable, is the statistical analysis and its interpretation appropriate?

Yes

Are all the source data underlying the results available to ensure full reproducibility?

Yes

Is the study design appropriate and is the work technically sound?

Yes

Are the conclusions drawn adequately supported by the results?

Yes

Are sufficient details of methods and analysis provided to allow replication by others?

Yes

Reviewer Expertise:

Neuropharmacology and oncology.

I confirm that I have read this submission and believe that I have an appropriate level of expertise to confirm that it is of an acceptable scientific standard.

# Peer review: Reviewer response for version 1

**DOI:** 10.5256/f1000research.136252.r150890

**Published:** 2022-10-03

**Authors:** Israa Albanaa

**Affiliations:** 1 College of Pharmacy, Department of Pharmacology and Toxicology, University of Mosul, Mosul, Iraq

The paper did good lab work, but lack clarity and consistency of the work.

 The citation is not accurate; the authors are citing the same paper which is under review.

 In the results section, the presentation of the data is not clear, in particular the comparison between different groups. It would be better if the authors specify the compared group in the graphs so the reader can easily follow the results.

 The discussion is not thorough; it would be more beneficial if they indicate the significance of the GSH and MDA so the reader could relate to the subject. I would recommend accepting the article after major corrections.

 Please refer to the annotated pdf for additional notes, located here.

Is the work clearly and accurately presented and does it cite the current literature?

Partly

If applicable, is the statistical analysis and its interpretation appropriate?

Partly

Are all the source data underlying the results available to ensure full reproducibility?

Partly

Is the study design appropriate and is the work technically sound?

Yes

Are the conclusions drawn adequately supported by the results?

Yes

Are sufficient details of methods and analysis provided to allow replication by others?

Yes

Reviewer Expertise:

Pharmacology and toxicology

I confirm that I have read this submission and believe that I have an appropriate level of expertise to confirm that it is of an acceptable scientific standard, however I have significant reservations, as outlined above.

# Response

**Published:** 2022-10-12

**Authors:** rana qais

**Affiliations:** pharmacist, Iraq

Thank you Dr. Israa for review my article, I made changes according to the notes that you sent on pdf and made a new version of the article.

 About the (enzymatic method) section I wrote the procedure according to the journal instructions include the detail of the method used of the laboratory analysis.

 I moved the figure (1) to the result section.

 I changed the wrong references but the reference no 37,38,39 remain because these references include the data of result (data of GSH, MDA, urea and creatinine also the data of histopathological score and the other histological images) and refer to this data according to the journal instruction of and put this data on repository such as zenodo.

 I can’t change the method of comparing in the result section, I made it according to my supervisor in the study.

 I added some explanation on the MDA and GSH in the discussion section

# Peer review: Reviewer response for version 1

**DOI:** 10.5256/f1000research.136252.r149805

**Published:** 2022-09-20

**Authors:** Hasan Alhaddad

**Affiliations:** 1 Moffitt Cancer Center, Toledo, OH, USA

This interesting manuscript studies the possible effect of cyanocobalamin to protect from methotrexate induced nephrotoxicity. Authors showed that cyanocobalamin protected rat kidneys from methotrexate toxicity through reduction of oxidative stress, increase GSH and reduction in lesions.

 This paper would be more valuable and interesting for readers if the authors revise the manuscript and address the following issues:

 1. The overall writing style of the paper needs more attention. I would recommend to improve the writing and correct some grammar and punctuation errors.

 2. The rationale for use of cyanocobalamin as a protective agent is not clear in the introduction. Is it utilized due to its antioxidant action? Please add some justification for why you chose this drug, i.e. add some previous studies that support its value as a possible protective agent.

 3.  In the methods, group II treatment is not clear. Please rephrase the treatment paradigm for this group.

 4. The figures need more attention. Please make sure to have a unified x axis title. Please mention what the letters a, b, c, and d on the bars represent. Try to expand the figure legends to accurately describe the figure contents in details. Also, make sure to not cut part of the axis titles.

 5. Again, revise all the writing. Please make sure to not miss words, for example: *Although MDA levels in renal tissue homogenate (P<0.05) significantly in the IV & V groups, and decreased significantly in group VI compared to the the MTX treated group (P<0.01); *In this case, is the MDA levels in renal tissue is higher or lower compared to IV and V groups. Please revise all.

Is the work clearly and accurately presented and does it cite the current literature?

Yes

If applicable, is the statistical analysis and its interpretation appropriate?

Yes

Are all the source data underlying the results available to ensure full reproducibility?

Yes

Is the study design appropriate and is the work technically sound?

Yes

Are the conclusions drawn adequately supported by the results?

Yes

Are sufficient details of methods and analysis provided to allow replication by others?

Yes

Reviewer Expertise:

Neuropharmacology and oncology.

I confirm that I have read this submission and believe that I have an appropriate level of expertise to confirm that it is of an acceptable scientific standard, however I have significant reservations, as outlined above.

# Response

**Published:** 2022-10-11

**Authors:** rana qais

**Affiliations:** pharmacist, Iraq

Thank you Dr. Hasan for review of article, I made some change in the new version according to your notes.

 The answers to your notes:

 1. I made changes to these issues.

 2.In this study, vit B12 was selected due to antioxidant action, according to previous animal studies, in which vitamin B12 can lower oxidative stress when used with different drugs such as tamoxifen and gentamycin. Vit B12 act as antioxidant by different mechanism: as a direct superoxide scavenger; in addition, may indirectly enhance ROS scavenging by protecting glutathione, which probably involves a complex network of processes that is not yet fully understood

 3. I rephrased (7 rats were treated with   a single IP dose of methotrexate (MTX) (20 mg/kg) followed by single IP injection of 0.5ml normal saline for 4 days. This group was served as positive control).

 4. I added this explanation (Values expressed in non-identical letters (a, b, c, d) are significantly difference (p<0.05).

 5. The MDA significant decrease *(P<0.05) *  in group IV, V and highly significant decreased in group VI compared to MTX treaded group II(P *<0.01). *I changed in the article (Although MDA levels in renal tissue homogenate (P<0.05) significantly decrease in the IV & V groups, and highly significant decrease in group VI compared to the MTX treated group (P<0.01).
